# Non-thermal plasma specifically kills oral squamous cell carcinoma cells in a catalytic Fe(II)-dependent manner

**DOI:** 10.3164/jcbn.18-91

**Published:** 2019-06-01

**Authors:** Kotaro Sato, Lei Shi, Fumiya Ito, Yuuki Ohara, Yashiro Motooka, Hiromasa Tanaka, Masaaki Mizuno, Masaru Hori, Tasuku Hirayama, Hideharu Hibi, Shinya Toyokuni

**Affiliations:** 1 Department of Pathology and Biological Responses, Nagoya University Graduate School of Medicine, 65 Turumai-cho, Showa-ku, Nagoya 466-8550, Japan; 2 Department of Oral and Maxillofacial Surgery, Nagoya University Graduate School of Medicine, 65 Tsurumai-cho, Showa-ku, Nagoya 466-8550, Japan; 3 Center for Advanced Medicine and Clinical Research, Nagoya University Hospital, Nagoya 466-8550, Japan; 4 Plasma Nanotechnology Research Center, Nagoya University, Nagoya 464-8603, Japan; 5 The Laboratory of Pharmaceutical and Medicinal Chemistry, Gifu Pharmaceutical University, Gifu 501-1196, Japan; 6 Sydney Medical School, The University of Sydney, NSW 2006, Australia

**Keywords:** non-thermal plasma, oral squamous cell carcinoma, catalytic ferrous iron, apoptosis, ferroptosis

## Abstract

Oral cancer accounts for ~2% of all cancers worldwide, and therapeutic intervention is closely associated with quality of life. Here, we evaluated the effects of non-thermal plasma on oral squamous cell carcinoma cells with special reference to catalytic Fe(II). Non-thermal plasma exerted a specific killing effect on oral squamous cell carcinoma cells in comparison to fibroblasts. Furthermore, the effect was dependent on the amounts of catalytic Fe(II), present especially in lysosomes. After non-thermal plasma application, lipid peroxidation occurred and peroxides and mitochondrial superoxide were generated. Cancer cell death by non-thermal plasma was promoted dose-dependently by prior application of ferric ammonium citrate and prevented by desferrioxamine, suggesting the association of ferroptosis. Potential involvement of apoptosis was also observed with positive terminal deoxynucleaotidyl transferase-mediated dUTP nick end labeling and annexin V results. Non-thermal plasma exposure significantly suppressed the migratory, invasive and colony-forming abilities of squamous cell carcinoma cells. The oral cavity is easily observable; therefore, non-thermal plasma can be directly applied to the oral cavity to kill oral squamous cell carcinoma without damaging fibroblasts. In conclusion, non-thermal plasma treatment is a potential therapeutic option for oral cancer.

## Introduction

Oral cancer accounts for approximately 2% of all cancers worldwide, but the proportion has gradually increased in some countries.^(1)^ Smoking, alcohol abuse, use of smokeless tobacco products and HPV infections are listed as high-risk factors for oral cancer.^(2–4)^ As oral cancer develops and infiltrates into the surrounding tissues with accompanying necrosis, it causes severe pain, nerve damage and facial deformation and is directly linked to a decrease in quality of life. Therefore, early detection and treatment are very important, as in other cancers. In addition, monitoring for locoregional recurrence and distant metastasis after surgery is also important.

Plasma is the fourth physical state beyond the normal solid/liquid/gas phases and is a mixture of gas, radicals, electrons, cations, anions, and ultraviolet (UV) radiation.^(5,6)^ Non-thermal plasma (NTP) represents an apparently contradictory condition with high energy but at near-body temperature, which was realized through engineering in the 1990’s. NTP is currently applied in preclinical experiments in a variety of fields, especially in the medical field.^(7–10)^ Studies have shown that NTP acts specifically on cancer cells, leading to cell death, and therefore represents a notable new potential cancer treatment.^(11–14)^ NTP has the same temperature as body temperature, and many chemical species/agents have been detected with NTP exposure, including ^•^OH, H_2_O_2_, O_2_^−^, nitric oxide, electrons and UV radiation.^(15)^ Sufficient evidence demonstrates that NTP promotes wound healing and possibly kills cancer cells.^(13,16–18)^ The theoretical basis for NTP use in cancer treatment is that cancer cells are oxidatively stressed in general, and the Fenton reaction occurs after NTP exposure.^(19)^

We have thus far conducted various research projects on the relationship between excess iron and carcinogenesis^(20–25)^ and recently showed that iron chelation or phlebotomy by reducing local iron can suppress mesothelial carcinogenesis.^(26,27)^ A phlebotomy intervention trial in humans led to not only a decreased cancer incidence but also significantly decreased cancer mortality;^(28)^ thus, a close relationship exists between cancer and iron. Although the effect of NTP on oral cancer cells has previously been reported,^(29,30)^ research has not been performed to explore the relationship between oral cancer cells and iron metabolism. Thus, the objective of this study was to evaluate the effect of NTP on oral cancer cells and its association with catalytic Fe(II).

## Materials and Methods

### Cell lines and reagents

We used seven human oral squamous cell carcinoma cell lines (SAS, Ca9-22, HSC-2, HSC-3, HSC-4, Sa3 and Ho-1-u-1) and two human fibroblast cell lines (HS-K and IMR-90-SV) in this study. The oral squamous cell carcinoma cells were all obtained from the Riken Cell Bank (Ibaraki, Japan). The ﬁbroblast cell lines were also obtained from the Riken Cell Bank and were used as a control. All the cells were grown in RPMI-1640 medium (Wako, Osaka, Japan) containing 10% fetal bovine serum (FBS) (Biowest, Nuaillé, France) and 1% antibiotic-antimycotic solution (Invitrogen, Carlsbad, CA), hereafter referred to as complete medium, and 0.25% trypsin (Nacalai Tesque, Kyoto, Japan) was used to collect cells. The cells were maintained at 37°C in a humidiﬁed incubator with 5% CO_2_.

### Non-thermal plasma (NTP)

NTP was generated by the following well-established procedure^(17)^ using a Habahiro (wide oriﬁce) instrument (from Prof. M. Hori, Plasma Nanotechnology Research Center, Nagoya University, Nagoya, Japan) by applying 10 kV from a 60-Hz commercial power supply, using 2 electrodes that were 20 mm apart. The NTP had an ultrahigh electron density (approximately 2 × 10^16^ cm^3^) and an O density of approximately 4 × 10^15^ cm^3^, as previously reported.^(31)^ Argon (Ar) was used as the gas to generate NTP and was implemented at a ﬂow rate of 2 L/min. The distance between the plasma source and the samples was ﬁxed at L = 8 mm. In all the experiments, cells in complete media were treated with NTP for 30–120 s at 25°C and then returned to 37°C for various incubation periods. For studies using 96-well plates, a cover was placed to expose only one well, as previously reported.^(31)^ As a relevant control, studies were performed using Ar alone under the same experimental conditions.

### Cell viability assay

Nine kinds of cell lines were cultured in 96-well plates (Thermo Fisher Scientiﬁc, Waltham, MA) at 5,000 cells/well for 24 h at 37°C. NTP exposure (30, 60, 90, and 120 s at 25°C) or Ar gas only was applied as a control to the cells, which were then cultured for 24 h at 37°C. Absorbance was measured at 450 nm using the WST-8 cell proliferation assay (Nacalai Tesque) and the POWERSCAN 4 microtiter plate reader (BioTek, Winooski, VT) to determine the viability of the cells after the treatment.

### Annexin V-fluorescein isothiocyanate (FITC) and propidium iodide (PI) analyses and terminal deoxynucleotidyl transferase-mediated dUTP nick end labeling (TUNEL) assay

We selected two squamous cell carcinoma cell lines (SAS and Ca9-22) that were highly sensitive to NTP and one non-tumorous fibroblast line (IMR-90-SV). In the NTP group and the Ar alone group, a total of 1.5 × 10^6^ cells/well were seeded in a 60-mm dish (Corning, Corning, NY) and cultured for 24 h. NTP or Ar alone was applied, and the cells were incubated for an additional 24 h. After incubation, we used the Annexin V-FITC Apoptosis Detection Kit according to the manufacturer’s protocol (Nacalai Tesque) and evaluated cell death using the Gallios flow cytometer (Beckman Coulter, Brea, CA). Furthermore, we performed a TUNEL assay. In the NTP group and the Ar alone group, a total of 5,000 cells/well were seeded in a 96-well plate (Thermo Fisher Scientiﬁc) and cultured for 24 h. NTP or Ar alone was applied, and the cells were incubated for another 24 h. After incubation, we used the In Situ Cell Death Detection Kit, Fluorescein to observe apoptosis microscopically according to the manufacturer’s protocol (Roche Applied Science, Mannheim, Germany) and observed the staining results, using the BZ9000 microscope (Keyence, Osaka, Japan).

### Quantitation and localization of catalytic Fe(II)

SiRhoNox-1, which is a catalytic Fe(II)-specific probe, was synthesized and characterized as previously described^(32)^ and was adjusted to 1 µM in RPMI-1640 without phenol red (Wako). Then, 5 × 10^5^ cells/well were seeded in a 60-mm dish and cultured for 24 h. After incubation, the cells were stained with SiRhoNox-1 (1 µM) for 30 min and washed with phosphate-buﬀered saline (PBS) (Wako). The fluorescence intensity of each cell was evaluated using a Gallios flow cytometer. Subsequently, 2.5 × 10^5^ cells were plated into glass-bottom dishes (Matunami, Osaka, Japan) and incubated overnight. Then, the cells were stained with SiRhoNox-1 (1 µM), MitoTracker Green FM (50 nM: Thermo Fisher Scientiﬁc) and ER-Tracker Blue-White DPX (200 nM: Thermo Fisher Scientiﬁc) for 30 min and LysoTracker Red DND-99 (200 nM: Thermo Fisher Scientiﬁc) for 60 min. The cells were then washed twice with PBS and observed using a Zeiss confocal microscope LSM880 (Carl Zeiss, Oberkochen, Germany). The fluorescence intensity for localization was evaluated, using ImageJ 4.7v software (National Institutes of Health, Bethesda, MD).

### Effects of ferric ammonium citrate (FAC) and desferrioxamine (DFO) administration prior to NTP application

SAS and Ca9-22 cells were seeded in 60-mm dishes at 5.0 × 10^5^ cells for 24 h. Ferric ammonium citrate (FAC) (10 µg/ml: Sigma-Aldrich, St. Louis, MO), which is a well-characterized cellular Fe donor, was dissolved in milli-Q water, and the cells were loaded for 3 h with FAC. Then, the culture medium was exchanged and stained with SiRhoNox-1 (1 µM) for 30 min and washed twice with PBS. Desferrioxamine (DFO) (25 µM: Sigma-Aldrich), which is known to decrease cellular iron levels, was dissolved in dimethyl sulfoxide, and the cells were loaded for 3 h with DFO. Then, the culture medium was exchanged and stained with SiRhoNox-1 (1 µM) for 30 min and washed twice with PBS. The fluorescence intensity of each cell was analyzed, using Gallios flow cytometry. Subsequently, SAS and Ca9-22 cells were cultured in 96-well plates at 5,000 cells/well for 24 h at 37°C. FAC was loaded into the cells for 3 h, and then the culture medium was exchanged. NTP was applied for 30 s, and the cells were cultured for 24 h at 37°C. DFO was also loaded into the cells for 3 h. Then, the culture medium was exchanged, NTP was applied for 90 s, and the cells were cultured for 24 h at 37°C. Absorbance was measured at 450 nm, using the WST-8 cell proliferation assay and POWERSCAN 4 microtiter plate reader to determine the viability of the cells after application.

### Analysis of reactive oxygen species (ROS) generation after applying NTP

In the NTP group and the Ar alone group, a total of 5.0 × 10^5^ cells/well were seeded in a 60-mm dish and cultured for 24 h. We removed the medium and added plain RPMI (FBS–, ANTI–). Then, NTP or Ar alone was applied for 60 s, followed by incubation for 30 min. We stained the cells with MitoSOX (5 µM: Thermo Fisher Scientiﬁc) and a chloromethyl derivative of 2',7'-dichlorodihydrofluorescein (DCF) (10 µM: Thermo Fisher Scientiﬁc) in either RPMI-1640 without phenol red for 10 min or BODIPY 581/591 C11 (BODIPY) (2 µM: Thermo Fisher Scientiﬁc) in RPMI-1640 without phenol red for 15 min. After washing twice with PBS, we evaluated ROS, using the Gallios flow cytometer.

### Scratch wound-healing assay

Cells were seeded in 35-mm dishes (Iwaki, Shizuoka, Japan) and cultured to 100% confluence. Scratches were made with 200-µl pipette tips, and the cells were photographed with a phase contrast microscope. Then, the Ar alone group and the NTP group were examined (60 s). After 24 h, we observed the cells with a phase contrast microscope.

### Transwell migration assay

In the NTP group and the Ar alone group, a total of 1.0 × 10^6^ cells/well were seeded in a 60-mm dish and cultured for 24 h. NTP or Ar alone was applied, and the cells were incubated for 30 min. Then, a cell culture insert (Corning) was placed in a 24-well plate, and cells were seeded at 5.0 × 10^5^ cells/200 µl in the same well. After 24 h, the cells were stained with hematoxylin and counted.

### Transwell invasion assay

In the NTP group and the Ar alone group, a total of 1.0 × 10^6^ cells/well were seeded in a 60 mm dish and cultured for 24 h. After incubation, NTP or Ar alone was applied, and the cells were incubated for 30 min. Then, Matrigel matrix (Corning) was added to a cell culture insert, which was placed in a 24-well plate, and the cells were seeded at 5.0 × 10^5^ cells/200 µl, as previously reported.^(33)^ After 24 h, the cells were stained with hematoxylin and counted.

### Western blotting

For the treatments with NTP and Ar alone, a total of 1.0 × 10^6^ cells/well were seeded in a 60-mm dish and cultured for 24 h. After incubation, NTP or Ar alone was applied, and the cells were incubated for 24 h. Protein extraction and sodium dodecyl sulfate-polyacrylamide gel electrophoresis were performed, as previously described,^(34)^ using anti-matrix metalloproteinase-2 (MMP-2) rabbit polyclonal antibody (Cell Signaling Technology, G657; Danvers, MA) at 1:1,000 dilution.

### Soft agar colony formation assay

In the NTP group and the Ar alone group, a total of 1.0 × 10^6^ cells/well were seeded in 6-well plates (Corning) and cultured for 24 h. After incubation, NTP or Ar alone was applied, and the cells were incubated for 30 min. During incubation, we prepared agar, using Agar Noble (Becton, Dickinson and Company, Franklin Lakes, NJ). We poured 2.5 ml of 0.75% agar into the 6-well plate as bottom agar. After confirming that the bottom agar had solidified, we collected cells and poured 1.5 ml of 0.36% agar into the 6-well plate as top agar which was mixed in each well. We incubated the cells for 1 or 2 weeks and counted the number of colonies.

### Statistical analysis

Statistical analysis was performed using Student’s *t* test and one-way analysis of variance with GraphPad Prism 5 software (GraphPad Software, La Jolla, CA). The data are expressed as the means ± SE of the mean (*n* = 3–6) unless otherwise specified. All experiments were performed in triplicate (*p<0.05, **p<0.01, ***p<0.001).

## Results

### The killing effect of NTP is specific to squamous cell carcinoma cells

**Fig. 1:**
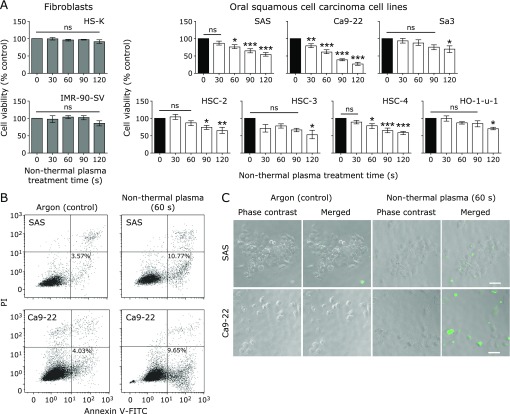
Cell viability assay and detection of apoptosis. (A) Cell viability assay. Nine cell lines were cultured in 96-well plates at 5,000 cells/well for 24 h at 37°C. Non-thermal plasma (NTP) (30, 60, 90, and 120 s at 25°C) was applied, Argon (Ar) alone was applied for 60 s to the control cells, and the cells were cultured for 24 h at 37°C. Absorbance was measured using the WST-8 cell proliferation assay and POWERSCAN 4 microtiter plate reader. Error bars represent the standard error of the mean. *p<0.05, **p<0.01, ***p<0.001; ns, not significant. (B) Annexin V-fluorescein isothiocyanate (FITC) and propidium iodide (PI) analysis. In the NTP group and the Ar alone group, a total of 1.5 × 10^6^ cells/well were seeded in a 60-mm dish and cultured for 24 h at 37°C. NTP or Ar alone was applied, and cells were incubated for 24 h at 37°C. After incubation, apoptosis was measured using flow cytometry. (C) Terminal deoxynucleotidyl transferase-mediated dUTP nick end labeling (TUNEL) assay. In the NTP group and the Ar alone group, a total of 5.0 × 10^3^ cells/well were seeded in a 96-well plate and cultured for 24 h at 37°C. NTP or Ar alone was applied, and cells were incubated for 24 h at 37°C. After incubation, a TUNEL assay was performed. Scale bar = 50 µm.

We performed a cell viability assay to evaluate the effect of NTP and compared differences in the effect between fibroblasts and squamous cell carcinoma cells. The results showed that the viability of HS-K and IMR-SV-90 cells did not significantly decrease upon NTP application until 120 s. However, the cell viability of the squamous cell carcinoma cell lines began to rapidly and significantly decrease with increasing NTP application time (Fig. 1A). SAS, HSC-3 and HO-1-u-1 cells are poorly differentiated cancer cells, whereas the other squamous cell carcinoma cells are well differentiated. The average application time leading to a significant decrease in cell survival was 100 s and 75 s, respectively, for each differentiation group. The exposure time until cell death by NTP for the well-differentiated cancer cells tended to be shorter than that for the poorly differentiated cancer cells. To evaluate cell death type of oral squamous cell carcinoma, we measured Annexin V-FITC and propidium iodide (PI) staining. We found that apoptosis occurred after NTP application to SAS and Ca9-22 cells (Fig. 1B). In addition, according to the TUNEL assay, we also observed apoptotic cells in the NTP group (Fig. 1C).

### Squamous cell carcinoma cells harbor more catalytic Fe(II) than fibroblast cells

**Fig. 2:**
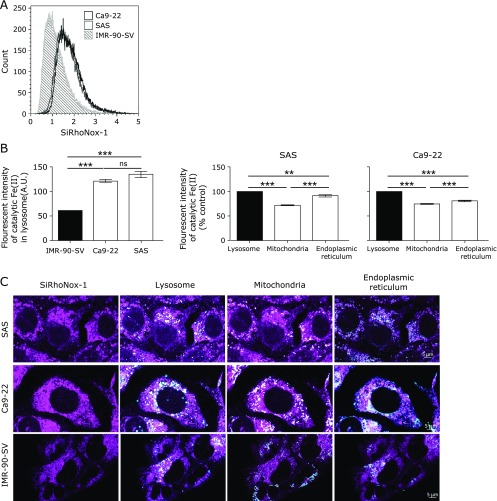
Squamous cell carcinoma cells contain more catalytic Fe(II) than fibroblast cells. (A) 5 × 10^5^ cells were stained with SiRhoNox-1 (1 µM), and the fluorescence intensity of individual cells was evaluated using flow cytometry. (B) A total of 2.5 × 10^5^ cells were plated into glass-bottom dishes and incubated for 24 h at 37°C. Then, the cells were stained with SiRhoNox-1 (1 µM), MitoTracker Green FM (50 nM), ER-Tracker Blue-White DPX (200 nM) and LysoTracker Red DND-99 (200 nM), and the cells were observed using a confocal microscope. Scale bar = 5 µm. Error bars represent the standard error of the mean (*n*>20). **p<0.01, ***p<0.001; ns, not significant.

To study the association between Fe and NTP cytotoxicity, we measured catalytic Fe(II) in individual cells using SiRhoNox-1. We observed that the Fe level in IMR-SV-90 fibroblast cells was lower than those in SAS and Ca9-22 cancer cells; however, no difference in the iron levels were observed between SAS and Ca9-22 cells according to the flow cytometric analysis (Fig. 2A). Consequently, to evaluate the localization of catalytic Fe(II), we observed the cells, using confocal microscopy. This examination revealed that the intensity of catalytic Fe(II) in the lysosomes of IMR-SV-90 cells was lower than that in SAS and Ca9-22 lysosomes (Fig. 2B). Furthermore, we found that the intensity of catalytic Fe(II) in lysosomes of cancer cells was higher than that in the mitochondria and endoplasmic reticulum (ER).

### The killing effects of NTP depend on the amounts of catalytic Fe(II)

**Fig. 3:**
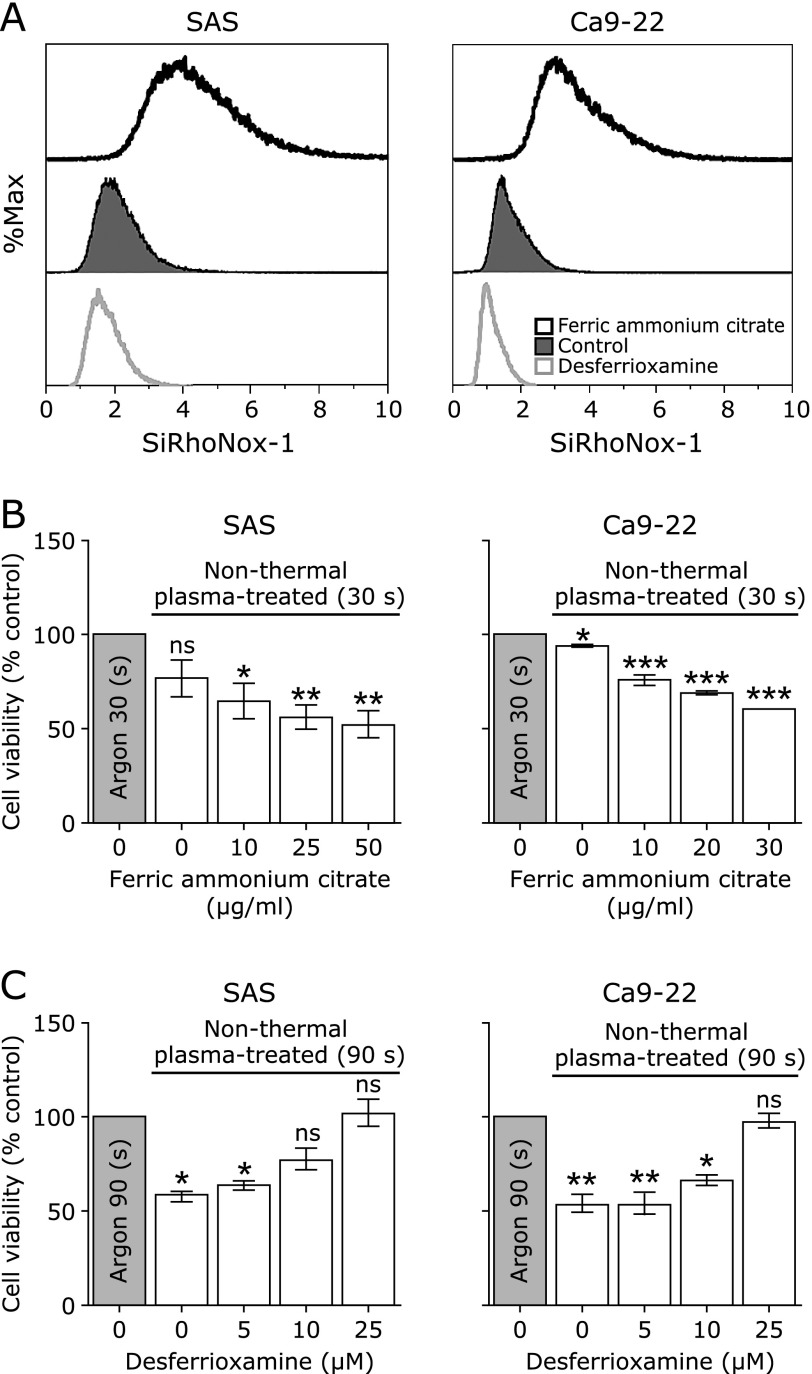
The effect of non-thermal plasma (NTP) changes depending on the amount of catalytic Fe(II). (A) SAS and Ca9-22 cells were seeded in 60-mm dishes at 5.0 × 10^5^ cells for 24 h. The cells were loaded for 3 h with ferric ammonium citrate (FAC) (10 µg/ml) and desferrioxamine (DFO) (25 µM) in each group. Then, the cells were stained with SiRhoNox-1 (1 µM) for 30 min. The fluorescence intensity of each cell was evaluated using flow cytometry. (B) SAS and Ca 9-22 cells were cultured in 96-well plates at 5,000 cells/well for 24 h at 37°C. The cells were loaded for 3 h with FAC. Then, NTP was applied for 30 s, and the cells were cultured for 24 h at 37°C. Absorbance was measured. (C) The cells were loaded with DFO for 3 h. Then, NTP was applied for 90 s, and the cells were cultured for 24 h at 37°C. Absorbance was measured. Error bars represent the standard error of the mean. *p<0.05, **p<0.01, ***p<0.001; ns, not significant.

We examined differences in the killing effect of NTP by modifying the intracellular iron level in each cell. SAS and Ca9-22 cells were loaded by FAC, and then the catalytic Fe(II) levels increased in both cell types. Conversely, when DFO was applied, the catalytic Fe(II) levels decreased in both cell types (Fig. 3A). Subsequently, we evaluated the effect of iron modification at NTP application. The results revealed that the viability of SAS and Ca9-22 cells significantly decreased as the applied concentration of FAC increased (Fig. 3B). Conversely, the viability of SAS and Ca9-22 cells significantly increased as the applied concentration of DFO increased (Fig. 3C).

### NTP exposure increases ROS in squamous cell carcinoma cells

**Fig. 4:**
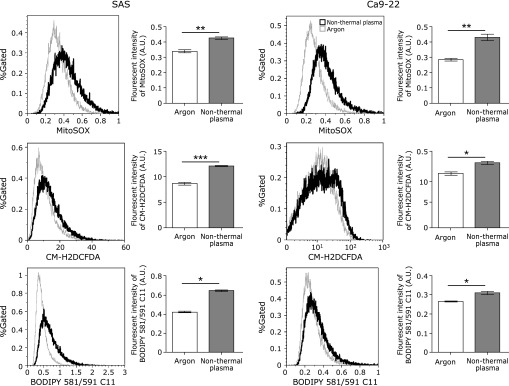
Non-thermal plasma results in reactive oxygen species (ROS) generation. ROS generation was detected using MitoSOX (5 µM), a chloromethyl derivative of 2',7'-dichlorodihydrofluorescein (DCF) (10 µM) and BODIPY 581/591 C11 (BODIPY) (2 µM) by flow cytometry. All signals significantly increased. Error bars represent the standard error of the mean. *p<0.05, **p<0.01, ***p<0.001; ns, not significant.

To evaluate ROS generation after applying NTP to squamous cell carcinoma cells, we used DCF, MitoSOX and BODIPY. DCF has been reported to detect various cytoplasmic peroxides, MitoSOX detects mitochondrial superoxide, and BODIPY reports membrane lipid peroxidation. After applying NTP, the level of ROS significantly increased for all the probes (Fig. 4).

### NTP suppresses the migration and invasion activities of squamous cell carcinoma cells

**Fig. 5:**
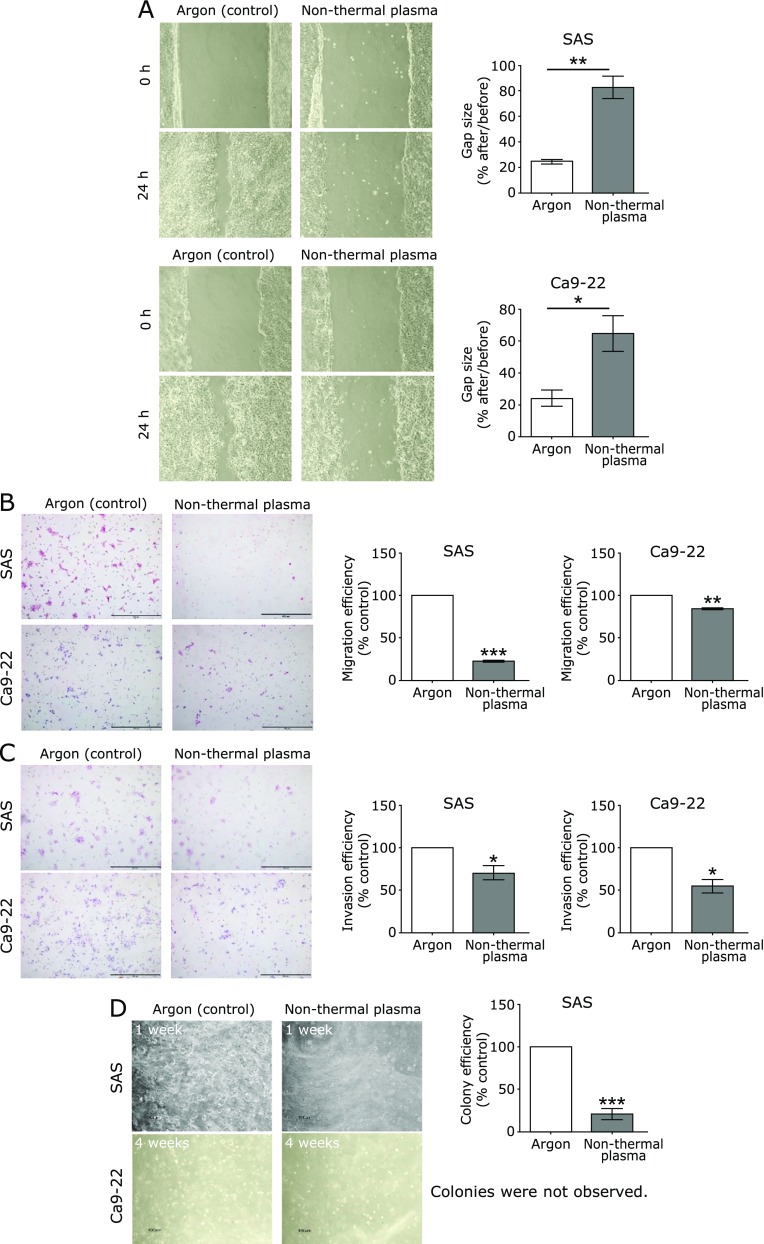
Non-thermal plasma suppresses the migration and invasion abilities of squamous cell carcinoma cells. (A) Wound-healing assay at 0 and 24 h after treatment. (B, C) Transwell migration and invasion assays at 24 h after treatment. Five representative fields of cells in each group were captured and analyzed. Scale bar = 500 µm. (D) Colony formation assay. Four representative fields of cells in each group were captured and analyzed. Scale bar = 500 µm. Error bars represent the standard error of the mean. *p<0.05, **p<0.01, ***p<0.001; ns, not significant.

To examine migration ability, we performed a scratch wound-healing assay and a transwell migration assay. The results revealed that NTP application suppressed the migratory ability of SAS and Ca9-22 cells compared to Ar alone, as shown in both assays (Fig. 5A and B). To evaluate the invasive nature of squamous cell carcinoma cells, we performed a transwell invasion assay. The results showed that NTP exposure suppressed the invasive ability of SAS and Ca9-22 cells compared with Ar alone (Fig. 5C). According to the Western blotting, the protein level of MMP-2 decreased in NTP group compared with Ar group (Supplemental Fig. 1*). Subsequently, the anchorage-independent proliferation of squamous cell carcinoma cells was assessed with a colony formation assay. The results revealed that for the SAS cell line, the Ar alone group showed significant anchorage-independent growth compared with the NTP group. However, in the case of the Ca9-22 cell line, colonies were not established in either the NTP or Ar alone group (Fig. 5D).

## Discussion

Here, we studied the effects of NTP exposure on oral squamous cell carcinoma for the first time, with a focus on catalytic Fe(II). A distinct difference in the effects of NTP was observed between the oral cancer cells and non-tumorous cells. We found a proportional association between the killing effect of NTP exposure and the amounts of catalytic Fe(II) in oral squamous cell carcinoma cells in iron modification experiments.

Oxidative stress via excess iron has been associated with carcinogenesis,^(20)^ and cancer cells are oxidatively stressed in general.^(35)^ In a previous experiment, we showed that cancer cells have more catalytic Fe(II) through maturation of HL-60 leukemia cells to macrophage-like cells.^(34)^ Here we revealed that abundance of catalytic Fe(II) was also true for the oral squamous cell carcinoma cells, with a higher fraction in the lysosomes than in the mitochondria and ER. In this experiment, we did not find a difference in the levels of catalytic Fe(II) between well-differentiated and poorly-differentiated oral cancer cells. However, we believe that this finding is the basis for the differential effect of NTP between tumor and non-tumor cells.

Regarding the type of cell death induced by NTP, we obtained a few evidences supporting apoptosis; namely, increased annexin V with flow cytometric analysis and TUNEL positivity. However, simultaneously, we observed iron-dependent lipid peroxidation and mitochondrial superoxide during cell death by the use of three different fluorescent probes, thus suggesting the mixed presence of ferroptotic process. In the case of mesothelioma cells exposed to NTP, we observed activation of the autophagic pathway and lipid peroxidation, suggesting ferroptosis.^(19)^ On the contrary, ovarian cancer and melanoma skin cancer cells mainly showed apoptosis upon NTP exposure.^(17,36)^ Therefore, the cell death type may be different among different cancer cells. Whether we can clearly differentiate between apoptosis and ferroptosis with the current techniques is an intriguing issue, which certainly requires further investigation, including development of novel methods to detect ferroptosis.

Due to the accessibility of the oral cavity, clinical application of NTP is easier at this site compared to other organs, except for the skin. The depth of penetration with direct NTP exposure is ~1 mm.^(15)^ We also showed that NTP treatment of oral cancer cells significantly impairs tumor migration and invasion, which may at least result from suppressed MMP-2 expression. As clinical applications, NTP can be used for preoperative progression control and irradiation of intraoperative margins rather than curative treatment. NTP treatment may be suitable for preventing recurrence after surgery. In general, a 10-mm safety margin is used in oral surgery. However, if this large margin can be reduced with NTP exposure, then functional loss can be reduced, resulting in cosmetic benefits and better quality of life. Notably, NTP exposure is substantially less harmful to normal tissues, as shown by the findings in fibroblasts, and can further promote wound healing.^(18,37)^ We believe that NTP exposure as an additional therapy is feasible until radiation therapy and chemotherapy are completed. Preclinical *in vivo* experiments are in progress to study the merits of NTP exposure.

In conclusion, NTP exposure specifically killed oral squamous carcinoma cells compared to fibroblasts, which was dependent on ample catalytic Fe(II). The type of cell death was mixed apoptosis and ferroptosis.

## Abbreviations

Ar: argon
BODIPY: BODIPY 581/591 C11
DCF: chloromethyl derivative of 2',7'-dichlorodihydrofluorescein
DFO: desferrioxamine (desferal)
ER: endoplasmic reticulum
FAC: ferric ammonium citrate
FITC: fluorescein isothiocyanate
FBS: fetal bovine serum
MMP-2: matrix metalloproteinase-2
NTP: non-thermal plasma
PBS: phosphate-buﬀered saline
PI: propidium iodide
ROS: reactive oxygen species
TUNEL: terminal deoxynucleotidyl transferase-mediated dUTP nick end labeling
UV: ultraviolet
