# Supplementary material for: Non-thermal plasma specifically kills oral squamous cell carcinoma cells in a catalytic Fe(II)-dependent manner
Source: J Clin Biochem Nutr. 2019 Jun 1;65(1):8–15. doi: 10.3164/jcbn.18-91 (PMC6667380; doi:10.3164/jcbn.18-91)
Supplement: Supplemental Figure 1 [file jcbn18-91sf01.pdf]

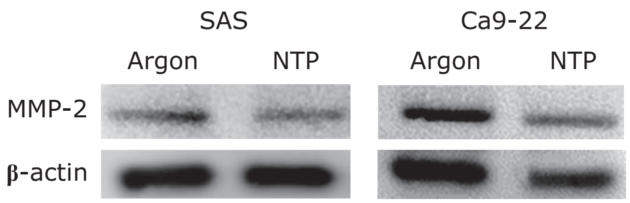

Supplemental Fig. 1. Non-thermal plasma (NTP) inhibits the tumor invasive activity of cancer cells. Matrix metalloproteinase-2 (MMP-2) was used to assess the invasive activity of cancer cells after the application of NTP. The protein level of MMP-2 was decreased in the NTP group compared with the Argon group.
